# Meningococcal B Vaccination (4CMenB) in Infants and Toddlers

**DOI:** 10.1155/2015/402381

**Published:** 2015-08-17

**Authors:** Susanna Esposito, Claudia Tagliabue, Samantha Bosis

**Affiliations:** Pediatric Highly Intensive Care Unit, Department of Pathophysiology and Transplantation, Università degli Studi di Milano, Fondazione IRCCS Ca' Granda Ospedale Maggiore Policlinico, 20122 Milan, Italy

## Abstract

*Neisseria meningitidis* is a Gram-negative pathogen that actively invades its human host and leads to the development of life-threatening pathologies. One of the leading causes of death in the world, *N. meningitidis* can be responsible for nearly 1,000 new infections per 100,000 subjects during an epidemic period. The bacterial species are classified into 12 serogroups, five of which (A, B, C, W, and Y) cause the majority of meningitides. The three purified protein conjugate vaccines currently available target serogroups A, C, W, and Y. Serogroup B has long been a challenge but the discovery of the complete genome sequence of an MenB strain has allowed the development of a specific four-component vaccine (4CMenB). This review describes the pathogenetic role of *N. meningitidis* and the recent literature concerning the new meningococcal vaccine.

## 1. Introduction

*Neisseria meningitidis* is one of the most frequent causes of meningitis and septicemia worldwide [1, 2], being not only responsible for 10–20% of specific meningococcal-related mortality but also the cause of (particularly pediatric) long-term morbidity [1] as it leads to permanent neurological* sequelae* and disabilities in an additional 20% [3–5]. Furthermore, the pediatric mortality rate among children with sepsis is over 20% [6, 7]. The incidence of meningococcal meningitis is greatest amongst children, adolescents, and adults aged up to 29 years, but young children are the most susceptible. The risk of* N. meningitidis* infection is particularly high in some regions of the world but, despite the introduction of innovations in health care, morbidity and mortality rates are high in both developed and undeveloped countries, and prevention is therefore a priority.

The bacteria colonise the nasopharyngeal tract of human hosts and are spread from subject to subject via air droplets. Transmission rates vary and are also related to individual risk factors such as age and/or underlying medical and social conditions (e.g., primary or secondary immunodeficiencies, a history of travel, and overcrowded living condition). Twelve different serogroups are known, but most invasive meningococcal diseases are caused by one of the six capsular groups A, B, C, W, X, and Y.

A number of excellent conjugate vaccines against serogroups A, C, W, and Y have been licensed, and the introduction of conjugate meningococcal C vaccine (MenC) has led to a rapid and sustained reduction in the incidence of invasive MenC disease across all age groups in Italy [8, 9]. However, a vaccine against capsular group B (MenB), which has now become responsible for most cases in Italy and the rest of the world [7, 9], has long eluded vaccinologists, particularly because of the problems associated with the B polysaccharide [10–12]. Unlike the highly immunogenic polysaccharides of serogroups A, C, W, and Y, the serogroup B polysaccharidic capsule contains a polysialic acid whose antigenic structure resembles the cell surface glycoproteins of human neurological tissue, and this has proved to be a formidable challenge [13].

The new protein-based vaccine against MenB (4CmenB; Bexsero, Novartis Vaccines and Diagnostics, Siena, Italy) has now overcome this barrier by using a cocktail of four main immunogenic components: two recombinant fusion proteins (*Neisseria* heparin-binding antigen [NHBA-GNA1030] and factor H binding protein [fHbp-GNA2091]), recombinant* Neisseria* adhesion A (NadA), and detergent-treated outer membrane vesicles (OMVs) derived from the NZ98/254 New Zealand meningococcal outbreak strain in which porin A (PorA 1.4) is the major immunodominant antigen. These components were identified using reverse vaccinology [14], a technique that analyses the whole bacterial genome in order to predict meningococcal antigens (exposed on the pathogen's surface or secreted) that can act as vaccine targets. NHBA is a surface b-barrel lipoprotein that binds to the anticoagulant heparin and induces protective immunity in human hosts [15–17], and fHbp is a surface-exposed protein that allows binding exclusively to human fH [18], mediates host serum resistance, and induces bactericidal antibodies upon host detection [15, 19]. NadA is a surface adhesin and invasin whose interactions with abundantly expressed human heat shock protein 90 (Hsp90) [20] also induce bactericidal antibodies [21, 22]. NadA expression can be regulated by the Nad repressor (NadR) [23]. Finally, porA (one of the two b-barrel porin proteins produced by* N. meningitides*) [24] assists in opsonophagocytic activity and is also involved in host actin reorganisation during infection, which depends on its ability to nucleate actin filaments.

This four-component meningococcal serogroup B vaccine (4CMenB), the first successful vaccine against the endemic form of this cause of serious bacterial meningitis and septicemia, has been in development for almost 20 years and has recently been approved for the active immunisation of subjects aged ≥2 months [9, 10] by licensing authorities in Europe, Canada, and Australia.

A bivalent fHbp recombinant vaccine (also known as LP2086; Trumenba, Pfizer Inc., Philadelphia, PA, USA) has been developed since 2006 and has now been approved by the US Food and Drug Administration for use in 10-to-25-year olds [25]. This vaccine appeared safe in a phase 3 study in approximately 5,600 healthy individuals 10 to 25 years of age and immunogenic and safe when coadministered with routine meningococcal A, C, Y, and W and tetanus, diphtheria, and pertussis (Tdap) vaccines in a phase 2 study in more than 2,600 healthy individuals 10 to 12 years of age [26]. Studies on this vaccine are ongoing in Europe and approval from European Medicines Agency is expected in 2017.

The aim of this review is to discuss the immunogenicity, safety, and tolerability of 4CMenB vaccine in infants and toddlers, and the efficacy of different vaccination strategies.

## 2. Immunogenicity of 4CMenB

An important challenge for the licensing of 4CMenB vaccine was the difficulty in showing its activity against epidemiologically relevant strains of* N. meningitidis* [27, 28]. After its development and phases I and II trials, some large-scale randomised phase III studies were planned in order to assess its efficacy and describe adverse reactions, but due to the rarity of the diseases caused by* N. meningitides* serotype B (which have annual rates of 0.5–5 per 100,000 people) laboratory-based methods were developed with the aim of predicting the vaccine's effectiveness and coverage [29]. In Europe, the vaccine was licensed on the basis of a correlate of protection calculated using a titre of human serum bactericidal activity (hSBA) that is present in convalescent patients which was shown to be protective in US Army recruits. HSBA assay is a recognised* in vitro* surrogate for evaluating protective immunity against* N. meningitidis*, and an adequate response is a crucial criterion for licensing vaccines against serogroup B meningococci. In order to justify the inclusion of each antigen in the formulation, it is necessary to run four hSBA assays on each serum sample, each using a* N. meningitidis* strain expressing the target antigen independently from the others in order to evaluate immunogenicity of each component of the vaccine, for example, a meningococcal strain that uniquely expresses NadA but not factor-H-binding protein or* Neisseria* heparin-binding antigen. Typically, an hSBA titre of 4 is initially used to assess immunogenicity. Subsequent phase III studies of 4CMenB in children used a more conservative titre of ≥5, which ensures that the level is >4 with 95% confidence taking into account within-assay variability.

The safety and immunogenicity of 4CMenB vaccine has been studied when administered at the same time as other routine infant vaccines (diphtheria, tetanus, acellular pertussis, inactivated poliovirus, hepatitis B,* Haemophilus influenzae* type b [DTaP-IPV-HepB/Hib], and 7-valent pneumococcal conjugate vaccine [PCV7]), and it has been found that the antibody responses to the routine vaccines are equivalent to those observed when the routine vaccines are given alone in the case of all of the antigens except for the pertactin component of acellular pertussis and pneumococcal serotype 6B [10]. However, this laboratory observation seems to be of no clinical significance, and published data also suggest that the incidence of pneumococcal disease due to serotype 6B is low in the countries in which PCV7 vaccination is used.

Other studies have investigated the persistence of bactericidal antibodies in young children after primary immunisation and the level of immunogenicity after a preschool booster [29, 30]. The levels of bactericidal antibodies after primary 4CMenB vaccination at the ages of two, four, six, and 12 months had waned when measured at 40–44 months, but an anamnestic response was observed following a booster dose given at the age of 40–44 months [14]. Similarly, bactericidal antibody levels in infants who originally received 4CMenB during late infancy (6, 8, and 12 months) had also waned when measured at the age of 40 months but, once again, there was an anamnestic response to a booster dose given at 40 months [30].

## 3. Safety and Tolerability of 4CMenB

The studies assessing the immunogenicity of 4CMenB also evaluated its safety and tolerability. The participating infants were observed for 30 minutes after each vaccine administration, and their parents were given a diary card on which to record the occurrence and severity of solicited local (i.e., injection site tenderness, erythema, induration, and swelling) and systemic reactions (i.e., changes in eating habits, sleepiness, vomiting, diarrhea, irritability, unusual crying, rash, and increased/decreased body temperature) and any other adverse events, during the following seven days. The rates of local and systemic reactions were similar to those seen following other routine infant and early childhood vaccinations, but injection site pain was consistently reported more frequently, especially by older children.

Fever was more frequent in the children who received 4CMenB together with other routine infant vaccines. It mainly occurred during the first 24 hours after administration but, as in case of other vaccines, it has been found that the prophylactic administration of paracetamol before and 4–6 hours after vaccination significantly reduces postvaccination fever without affecting immunological responses [31, 32].

The pivotal and phase IIb studies found that the most frequently reported local reaction of tenderness affected 87% of the 4CMenB injection sites, 80% of the DTaP- IPV-HepB/Hib sites, and 79% of the PCV7 sites when all three vaccines were administered together [33]. The frequency of reported tenderness after DTaP-IPV-HepB/Hib and PCV7 injections when they were administered without 4CmenB was respectively 59% and 53%, whereas when DTaP-IPV-HepB/Hib and PCV7 injections were administered with 4CmenB it was respectively 68% and 62%.

The reported rates of local reactions to 4CMenB were slightly higher than those related to routine vaccines [10], but the majority were transient, most intense on the day after vaccination, and resolved within a week.

Although the systemic reactions that occurred when 4CMenB was administered concomitantly with routine vaccines cannot be specifically attributed to one or other of the vaccines, it is possible to assess the overall profile. The occurrence of 4CmenB-related seizures is rare: the combined data of infant studies including >20,000 vaccinations in the primary 4CMenB study arm indicate an overall rate 0.1 febrile seizures/1000 vaccinations on the day of vaccination or the day after and no events in the control arm. They are similarly rare in toddlers: 0.4 events/1000 vaccinations (95% confidence interval (CI): 0.05–1.46) after a total of 11,000 4CMenB vaccinations administered with or without routine vaccines, as against 0.3 event/1000 visits (95% CI: 0.04–1.05) in the case of those receiving routine vaccines alone [10, 33].

Six suspected cases of Kawasaki disease reported during the course of two infant studies (four in the pivotal trial and two in the phase IIb study) were evaluated by an independent external expert panel in order to assess whether they were true Kawasaki cases and whether they were vaccine related [33]. Analysis of the Kawasaki cases indicates an annual incidence of 72/100,000 person-years (95% CI: 23–169) after 4CmenB vaccinations, as against 56/100,000 person-years (95% CI: 1–311) after routine vaccinations alone.

The overall data from different studies indicate that the frequency of febrile seizures, the incidence of Kawasaki disease, and the proportion of infants using antipyretics are similar to those observed during clinical licensure programmes. However, as the number of exposed infants is still too small to exclude any relationship with rare adverse events, further postmarketing surveillance is necessary.

## 4. Coverage and Efficacy of 4CMenB

The new 4CMenB vaccine may not protect against all invasive meningococcal B strains because the antigens included in the vaccine are expressed by only some of the strains in circulation. However, it is not known what protection 4CMenB vaccine provides against invasive meningococcal disease (IMD) because it depends on the vaccine antigens expressed by the meningococcal strains in any given geographical area and their cross-reactivity with the antigens included in the vaccine. Epidemiological and microbiological data regarding the circulating meningococcal strains are important in order to predict the theoretical coverage provided by 4CMenB vaccine and assess its impact on disease burden.

Further postmarketing surveillance will allow a more precise estimate of the effectiveness of 4CMenB. However, although an hSBA can be used to demonstrate whether the vaccine induces antibodies capable of killing meningococcal strains, the presence of four antigens means that it is more complex than in the case of other meningococcal vaccines (i.e., MenC). Moreover, the genetic diversity of serogroup B strains means that not all of them have the genes coding for each of the antigens, and their expression may vary over time or from place to place.

For these reasons, the Meningococcal Antigen Typing System (MATS) is used to measure bacterial antigen expression in order to predict whether bactericidal serum is capable of killing particular strains [34–36]. This method is characterised by both phenotypic and genotypic analyses: the expression of the individual antigens that cross-react with the corresponding vaccine antigen is quantified using polyclonal antibodies against NHBA, NadA, and fHbp in an enzyme-linked immunosorbent assay (ELISA), and DNA sequence homology to the variable region sequence of the vaccine strain PorA gene is assessed, in order to estimate coverage in a specific region [32, 33]. It has not yet been proved that there is a correlation between the MATS results and real vaccination coverage, but the predicted protection based on the expression of at least one matched antigen ranges from 73% to 87% [36]. The MATS has been applied to isolates of 1,052 MenB strains causing IMD in Europe submitted to reference laboratories in France, Germany, Italy, Norway, and the UK between 2007 and 2008 [37], and the analysis demonstrated estimated efficacy values ranging from 73% in the UK to 87% in Italy. Furthermore, a study conducted in Canada between 2006 and 2009 analysed 157 MenB isolates collected from children and adults with IMD and found that the potential coverage of 4CMenB vaccine was 75–90%. On the basis of the MATS ELISA findings, the authors predicted that 66% of the circulating strains were covered by at least one vaccine antigen although none were covered by all four [38].

A new meningococcal serotype X has recently been isolated in Africa, against which no vaccine is currently available. However, some authors have recently used the MATS and bactericidal assays of 11 serogroup X isolates taken from nine African and two French patients and found that 4CMenB vaccine could have a good coverage against the strains from Africa but not those from France [39]. In regions where meningococcal strains are appropriately monitored, the MATS can evaluate the real effectiveness of 4CmenB vaccine, and the development of changes in the MenB serogroup over time. It is also a very useful means of monitoring the emergence of new MenB mutants due to selective vaccination pressure [39].

## 5. Vaccination Strategies and Future Perspectives

Although the development of a meningococcal B serogroup vaccine was slow and difficult, the new Bexsero 4CMenB vaccine has recently been licensed in the EU, Australia, and Canada. The introduction of any new vaccine is never easy, but it is especially complex in this case.

The success of a vaccination programme is based on both cost-effectiveness and public acceptance and although meningococcal B infection is a cause for concern in the general population, the acceptability of the vaccine by parents is influenced by worries concerning its potential side effects, its real effectiveness, and the consequences of its coadministration with other routine vaccines in terms of the number of injections and possible immunological interference [40]. For example, its acceptance may be reduced by the fact that it has been associated with increased rates of fever when coadministered with the routine vaccinations provided during infancy on the basis of the national immunisation schedules.

One recent study of parental attitudes to 4CMenB showed that 82.5% of the interviewees wanted their children vaccinated. The most frequent concerns were side effects including fever (41.3%) and adequate vaccine testing (11.7%), but 26% of the parents said that they had no concerns. Moreover, as in the case of other vaccinations (e.g., HPV), the authors found that most parents (81.7%) were more likely to accept the vaccination if their immunisation providers recommended it [41].

The vaccination schedule should take into account the age of the subjects most frequently affected by meningococcal disease and the epidemiology of meningococcal infections. One recent study aimed at defining the optimal age for administering 4CMenB to children has shown that the incidence is highest in those aged <5 years (particularly those in their in the first year of life, when deaths are more frequent) and, on the basis of this finding, the authors suggested that vaccination should be started in the first year of life, with a catch-up dose being given at the age of five years [42].

Other recent studies have evaluated the cost-effectiveness of a potential MenB vaccination programme [43, 44]. One study carried out in The Netherlands estimated that an infant MenB vaccination programme would prevent 14% of cases over the lifetime of a birth cohort and concluded that this was not cost-effective [44] and although another study carried out in the UK estimated that routine vaccination would prevent 27–56% of cases over the lifetime of a birth cohort, the authors also considered it not cost-effective [41]. Finally, a recent study of the economic impact of MenB vaccination in Canada found that the MenB vaccination programme exceeds the generally cost-effectiveness thresholds and therefore should not be considered economically advantageous [45].

Nevertheless, 4CMenB is now being used in Canada and, upon parents' request, also in all of the countries and it has been licensed and its use in at-risk populations has been implemented. It has also been announced that it may be introduced into the UK's routine infant immunisation schedule using a 2 + 1 regimen although only a 3 + 1 regimen with the concomitant use of paracetamol is currently licensed [46], and some other countries are reconsidering their cost-effectiveness calculations by also considering its possible impact on carrier status [47].

With the availability of the bivalent rLP2086 vaccine for the adolescent age, it will be important to compare vaccination strategies that cover different age groups as well as to understand the impact of the two vaccines against IMD overall, meningococcal disease due to serogroups different from MenB and meningococcal carriage in the nasopharynx.

## 6. Conclusions

*N. meningitidis* is still one of the major causes of sepsis and meningitis among children worldwide and is associated with a high mortality rate. Considerable efforts have therefore been made to prevent meningococcal disease by means of vaccination, and two effective conjugate vaccines have recently been licensed and led to good results (Men C and MenACYW). However, it proved to be very difficult to develop a vaccine against serogroup B because of the poor immunogenicity of its capsular polysaccharide, even when conjugated with a carrier that could also induce an autoimmune response, until experts used reverse vaccinology to identify appropriate antigenic recombinant proteins. The overall findings of various studies have shown that the administration of three doses of 4CmenB to young children (alone or with routine vaccines) does not interfere with immune responses, and most have found that its safety and tolerability are acceptable. However, its coadministration with other vaccines does lead to increased reactogenicity (particularly fever) and so such coadministration should be combined with paracetamol given both before and after vaccination.

The new 4CMenB vaccine represents an important opportunity to fight pediatric IMDs, but its introduction should take into account the need to maintain the appropriate use of meningococcal conjugate vaccines that cover serogroups other than B, community opinion, and cost-effectiveness data. Moreover, it will be important to compare 4CMenB potential efficacy with that of bivalent rLP2086 vaccine. Finally, it is very important to continue surveillance in order to monitor the emergence of new meningococcal B strains in order to identify any that are not susceptible to 4CMenB.
